# Study on roadway layout and surrounding rock control of isolated island panel

**DOI:** 10.1038/s41598-023-46664-1

**Published:** 2023-11-10

**Authors:** Lei Shi, Jiao Zhang, Weiyong Lu, Dong Lv, Xiang Sun

**Affiliations:** 1 Department of Mining Engineering, Lyuliang University, Lvliang, 033001 Shanxi China; 2 School of Mining Engineering, Heilongjiang University of Science and Technology, Harbin, 150020 Heilongjiang China; 3 Lyuliang Engineering Research Center of Intelligent Coal Mine, Lvliang, 033001 Shanxi China; 4 School of Energy and Mining Engineering, China University of Mining and Technology (Beijing), Beijing, 100083 China; 5 Inner Mongolia Energy Group Co., Ltd., Hohhot, 010090 Inner Mongolia China; 6 Tongsheng Selian Coal Development Co., Ltd., Ordos, 014399 Inner MongoliaInner Mongolia China; 7 School of Civil Engineering and Architecture, Wuyi University, Wuyi, 354300 Fujian China

**Keywords:** Environmental impact, Natural hazards

## Abstract

During the re-mining of historical residual coal resources, the stress environment is complex, the surrounding rock conditions are bad, the mining roadway is significantly affected by ground pressure, the layout is difficult, and the safety is poor. Taking the recovery of isolated island coal pillar in 4# coal seam as the research background, based on the difference in the distribution morphology of the goaf on both sides of the isolated island coal pillar, the stress and failure law of the isolated island panel boundary are studied by numerical simulation method. (1) The peak stress difference of multiple goaf boundaries on both sides of the isolated island coal pillar is between 0.18 and 4.51 MPa. The peak stress is affected by the change of the length of the roof “cantilever beam” at the stopping line of the goaf, so that the peak stress of the goaf boundary is periodic. (2) The high stress is mainly concentrated in the center of the pillar. The peak stress at the end of each pillar is 35–40 MPa. The coal pillar bears high stress, and the stress zone of the original rock moves to the end of the coal pillar. (3) There is a plastic zone of 8–20 m at the corner of the end of each coal pillar. On the basis of the stress zone and failure zone distribution of the goaf boundary on both sides of the isolated island panel, the roadway layout of the isolated island panel is determined, that is, the air-return roadway of the isolated island panel is arranged at random, and the width of the isolated island coal pillar d1 is selected as 10 m. The transport roadway is arranged straight, and the transport roadway of the isolated island panel is in the width section area of the goaf X4103. The width d1 of the isolated island coal pillar is selected to be 8 m, and the length d5–d7 of the mining roadway layout in the width of the coal pillar is 24 m. The roadway of isolated island panel is divided into 4 areas for support control, and the drilling pressure relief technology is proposed for high stress roadway. Through the field monitoring data, it can be seen that the mining roadway can meet the requirements of isolated island coal pillar recovery, which provides reference for the layout and control of abandoned coal roadway in this mine and other mines.

## Introduction

Limited by historical conditions, technical level, mining planning and other factors, the phenomenon of “Mining easy coal seams instead of hard coal seams, Mining thick coal seams instead of thin coal seams” is widespread, resulting in a large amount of coal left over, resulting in a great waste of resources^1,2^. In addition, with the continuous increase of the depth and intensity of coal mining, the panel is frequently affected by strong dynamic pressure in the process of mining, which brings great hidden dangers to the safety of mine production^3–5^. At present, how to ensure the green, healthy and sustainable development of the coal industry is the focus of coal mines at this stage^6^. At the same time, the National Development and Reform Commission of China clearly put forward in the Interim Regulations on the Development and Utilization of Special and Scarce Coal: “China encourages production enterprises to carry out re-mining of special and scarce coal or mining of corner residual coal and extremely thin coal seams on the premise of safety, rationality and economy”^7^. At the present stage, the research on the coal pillar of the isolated island panel is mainly focused on the single goaf on both sides of the coal pillar, while the research on the goaf at different production stop locations with multiple heads on both sides of the coal pillar of the isolated island panel is less^8–10^. There are few studies on the stress distribution characteristics of the residual coal affected by the boundary of the goaf with different shapes and the layout of roadway, so the study of the on-site impact appearance and the stress distribution characteristics during the excavation has a better guiding significance for the prevention of rock burst during the mining of coal pillars, especially the determination and pretreatment of the stress anomaly area before mining in the panel. Based on 4# coal seam in residual island coal pillar recovery as the research background, system research island coal face of mined-out area and between goaf by multiple comprehensive coal pillar under the influence of the stress evolution, deformation and failure characteristics, puts forward an island coal pillar mining roadway arrangement, zoning control of surrounding rock and unloading technology, determine reasonable control parameters of surrounding rock.

## Engineering background

The mine is mainly mining No. 4 coal seam, the average depth of the working face is 408 m, the average thickness is 5.1 m, and the dip angle is 3°–5°. Fine sandstone with the immediate bottom thickness is 4–6.3 m, sandy mudstone with the immediate roof thickness is 2.7–3.3 m, siltite with the main roof thickness is 9–11 m. Four mined-out areas are distributed on both sides of the isolated island coal pillar of the coal seam, totaling eight 8 mined-out areas. The length of the isolated island coal pillar is 1380 m, and the width is 208–250 m. H1–H8 are the stopping lines of each goaf on both sides of isolated coal pillar. The spacing between the stopping lines of each goaf on both sides of the isolated coal pillar is based on the stopping lines H1 and H2. The spacing between the stopping lines H13, H15, H17, H24, H26 and H28 are respectively 12 m, 6 m, 0 m, 30 m, 6 m and 2 m. Z13 is the interval coal pillar between goaf X4101 and X4103. Similarly, the interval coal pillar also includes Z35, Z57, Z24, Z46 and Z68. The width of interval coal pillar Z13 is 32 m, and the width of other interval coal pillars is 24 m. The width of goaf X4101 in the collapse column area is 80 m, the width of goaf D4108 is 100 m, and the width of other goaf is 200 m. D1–D8 are the central position of the width of each goaf. There are 300 × 400 m surface high-voltage pylons near goaf D4102 to protect coal pillars.

**Figure 1:**
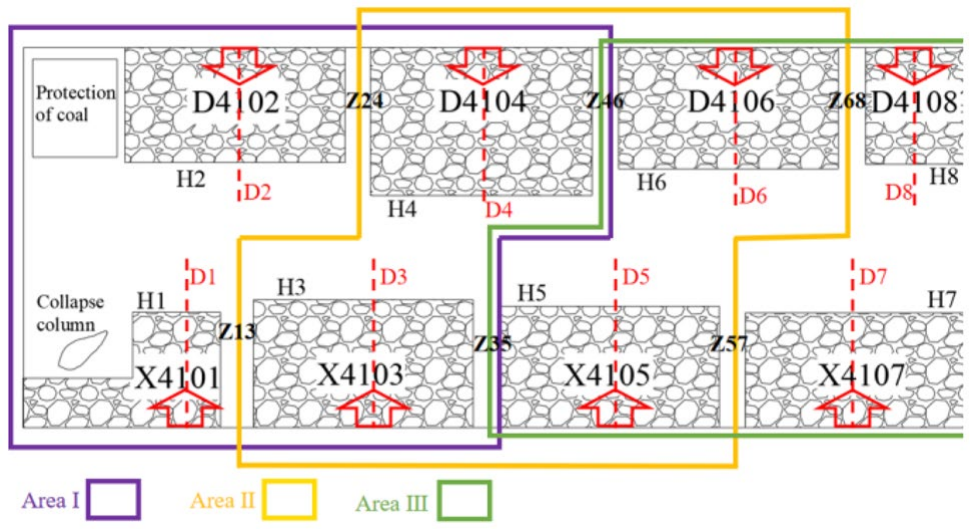
Plan of isolated island panel.

In recent years, in order to realize the sustainable development of the mine, the isolated island coal pillar recovery has been attempted. The air-return roadway is planned to be arranged on the D4102 side of the goaf, and the transport roadway is planned to be arranged on the X4101 side of the goaf. The premise of safe recovery of isolated island panel is reasonable layout of mining roadway. The distribution pattern of goaf on the side of air-return roadway is regular, but the spacing between stopping lines is relatively different. In order to achieve a higher coal recovery rate, return air roadway should be staggered layout. The difference of stopping lines on the side of the transport roadway is small. Considering the function of the transport roadway, the layout is straight. As shown in Fig. 1.

## Boundary stress and failure characteristics on both sides of isolated island panel

### Establishment of isolated island panel model

**Figure 2:**
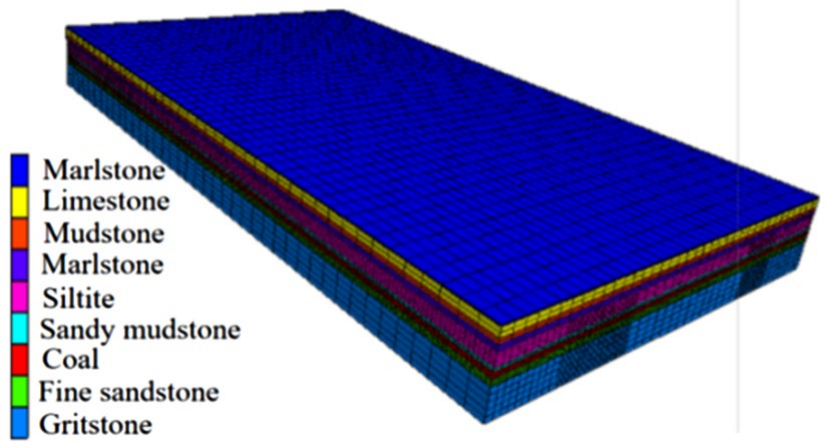
Numerical model.

Before mining, isolated island coal pillar is affected by several mining-induced stresses in the surrounding mined-out area. The stress environment and failure state of isolated island coal pillar are very important to the layout of mining roadway. According to the geological data of the mine, FLAC3D numerical simulation software was used to simulate the isolated island panel. Due to the huge model, the isolated island panel was simulated by areas, and the isolated island panel was divided into three regions, as shown in Fig. 1. The calculation models of the three areas were established respectively to simulate each goaf. Model dimensions length × width × height = 650 m × 500 m × 65 m, as shown in Fig. 2. An approximately equivalent uniform load is applied to the upper boundary, horizontal constraints are applied to the surrounding boundary, and the lower boundary is fixed. The *Mohr*–*Coulomb* yield criterion is adopted, and the mechanical parameters of rock mass are shown in Table 1.

**Table 1:** Mechanical parameters of rock mass.

Lithology	Bulk (GPa)	Shear modulus (GPa)	Internal friction (°)	Cohesion (MPa)	Tensile strengt h (MPa)	Density (kg/m^3^)
Marlstone	3.36	2.54	32	1.4	0.68	2540
Limestone	9.1	7.6	39	3.4	0.7	2450
Mudstone	5.9	4.2	28	1.8	0.8	2490
Marlstone	3.36	2.54	32	1.4	0.68	2540
Siltite	10.4	7.5	42	2.7	0.9	2570
Sandy mudstone	4.26	2.18	34	1.3	0.7	2510
Coal	2.42	1.31	25	0.8	1.6	1340
Fine sandstone	4.39	2.27	30	3.8	0.75	2500
Gritstone	8.05	5.9	48	3.1	0.8	2520

### Stress distribution at the boundary of each goaf on both sides of the isolated island panel

**Figure 3:**
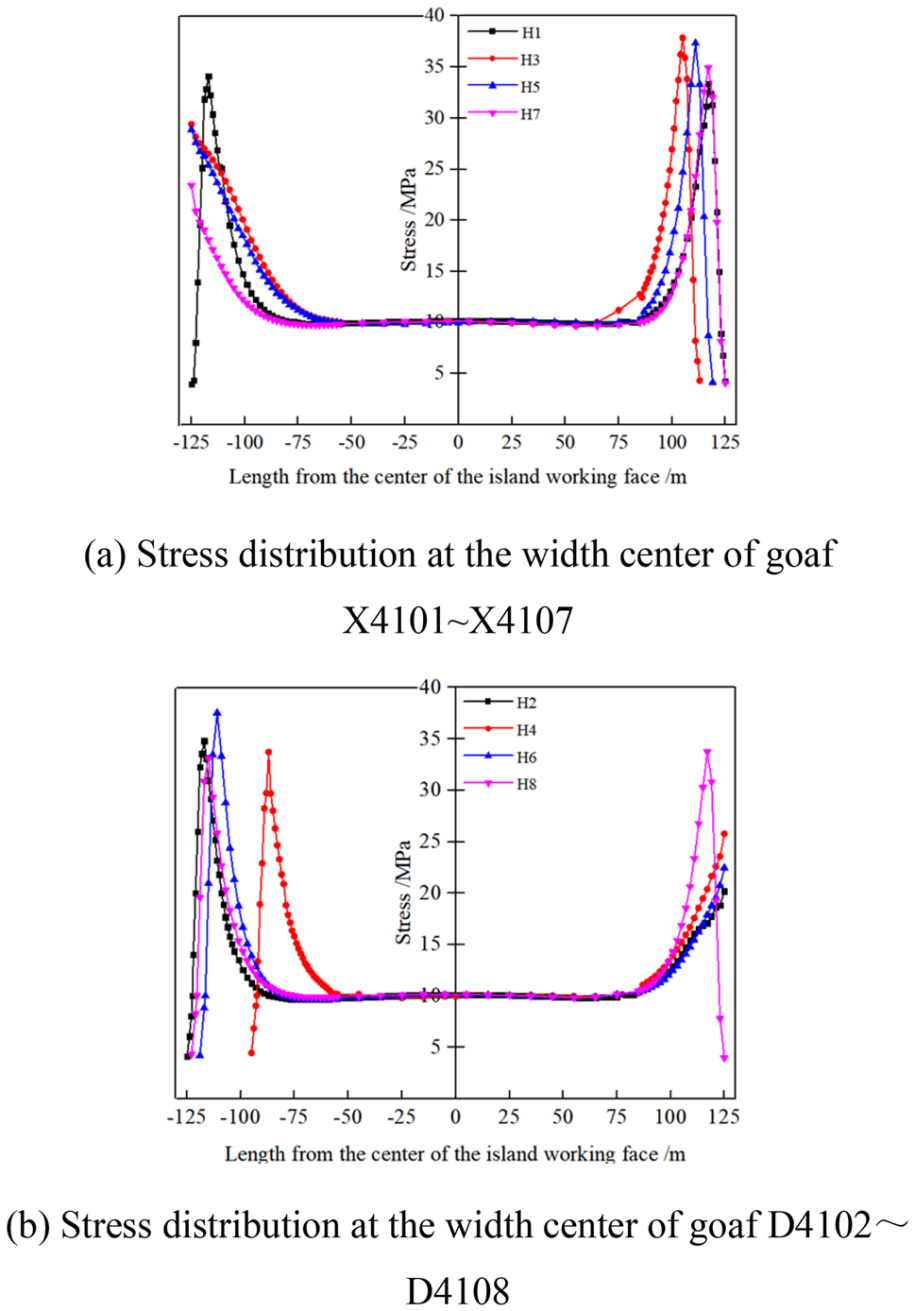
Stress distribution at different cross sections of the isolated island panel.

The working faces on both sides of the isolated island panel have been fully mined. The vertical stress distribution of the isolated island panel is shown in Fig. 3 at the cross section Di_(i=1–8)_ in the center of each goaf. The center of the isolated island panel is the origin, and the distance between the stopping lines H1 and H2 is 250 m at the widest point of the isolated island panel. The positive X axis is the direction of X4101–X4107 goaf, and the negative X axis is the direction of D4102–D4108 goaf.

Based on the numerical simulation of stress field distribution, and extract the value of the vertical stress around the isolated island panel analysis can be obtained. It can be seen from Fig. 3a that in the lateral direction of X4101 to X4107, the end of the isolated island panel is 2.3 m, 2.5 m, 2.4 m and 2.3 m away from the edges of stopping lines H1, H3, H5 and H7 in each goaf, respectively, which are stress reduction areas. The peak stress was located 9 m from H1, H3, H5 and H7 edges, and the peak stresses were 33.34 MPa, 37.85 MPa, 37.34 MPa and 34.92 MPa, respectively. The areas 40 m, 39 m, 40 m and 38 m away from the edges of H1, H3, H5 and H7 are original rock stress zone, and the stress value is 10.2 MPa.

Similarly, it can be seen from Fig. 3b that in the lateral direction of D4102–D4108, the end of the isolated island panel is 2.4 m, 2.5 m, 2.4 m and 2.4 m away from the edges of the stoppage lines H2, H4, H6 and H8 of each goaf, respectively, which is the stress reduction area. The peak stress is about 9 m from the edges of H2, H4, H6 and H8, and the peak stresses are 34.79 MPa, 33.71 MPa, 37.52 MPa and 33.08 MPa, respectively. The areas 38 m, 38 m, 38 m and 40 m away from the edges of H2, H4, H6 and H8 are original rock stress.

Therefore, the stress zoning range generated by each goaf at the edge of the isolated island panel is basically the same, that is, the range of stress reduction area, stress increase area and original rock stress area. There is a difference in the peak stress at the ends of both sides of the isolated island coal pillar, and the difference in the peak stress is 0.18–4.51 MPa. This is due to the periodic breaking of the “beam structure” above the roof during the mining process of the working face, the different positions of the stopping line in the goaf, and the different lengths of the "beam structure" of the roof of the stopping line. The peak stress of each goaf in the coal body at the ends of both sides of isolated island coal pillar increases first and then decreases with the position of stopping line Hi_(i= 1–8)_ deep into isolated island coal pillar, which is basically consistent with the periodic pressure step distance of 28–34 m in actual engineering.

### Stress distribution of coal pillars in each area on both sides of isolated island panel

**Figure 4:**
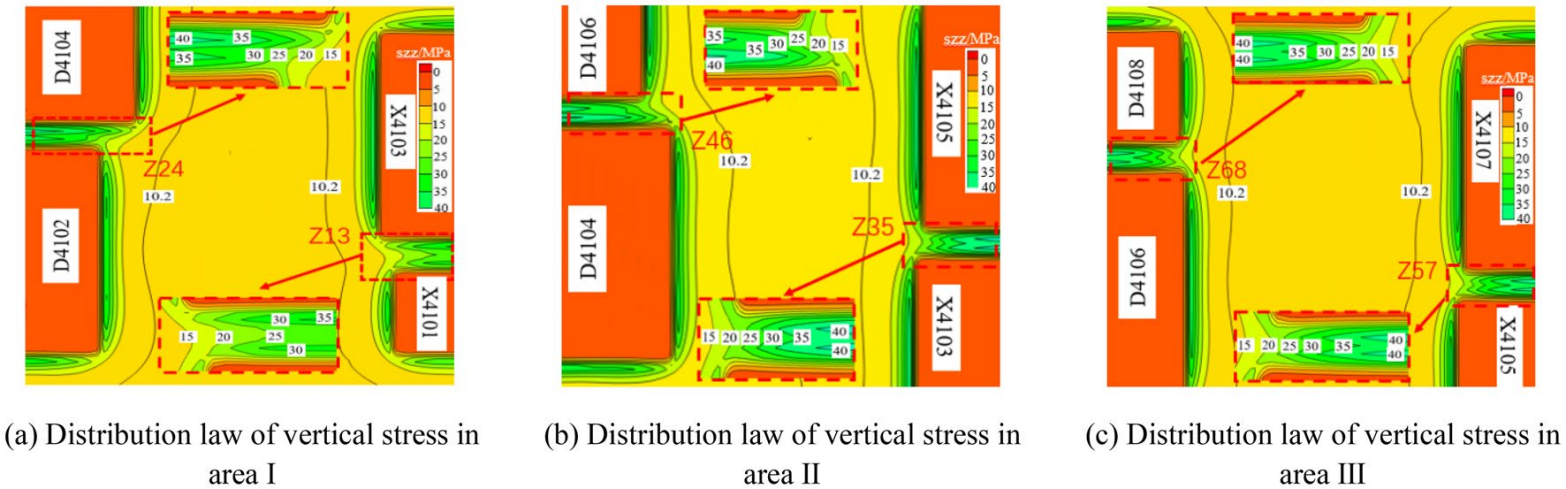
Stress distribution in coal pillar of working face.

As shown in Fig. 4, the stress distribution of coal pillars in areas between goafs on both sides of the isolated island panel after the stability of the isolated island panel is simulated by areas. Figure 4a–c shows the vertical stress distribution of section coal pillar area. The stopping line Hi_(i=1–7)_ of each goaf is parallel to but not aligned with the advancing direction of the isolated island panel. The section coal pillar between goaf is superimposed by the stress of goaf on both sides, and the high stress is mainly concentrated in the middle of the section coal pillar. The peak stress of each section coal pillar at the end of both sides of the isolated island panel is 35–40 MPa. The section coal pillar bears high stress and the stress zone of original rock stress zone moves to the side of section coal pillar.

### Failure characteristics of boundary in each area on both sides of isolated island panel

**Figure 5:**
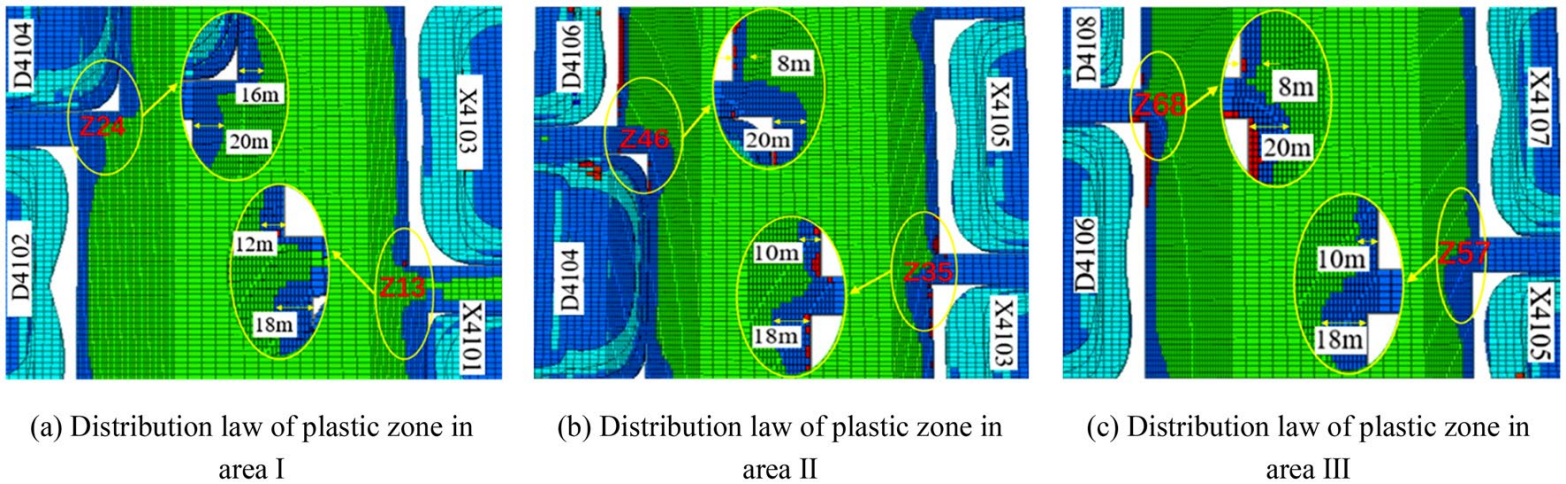
Plastic zone distribution of boundary in each area on both sides of isolated island panel.

As shown in Fig. 5, after the stability of each goaf, the plastic zone distribution of the goaf and coal pillar on both sides of the isolated island panel. It can be seen from Fig. 5a–c that there is an elastic zone of 4–16 m in section Z13, while there is no elastic zone in the remaining section and the coal pillar is in a plastic state. In the direction perpendicular to the advancing direction of the isolated island panel, there is a plastic zone of 8–20 m at the corner of the coal pillar end of each panel, which is larger than the plastic zone in the middle of each goaf. In this area, it is necessary to strengthen the support of the roadway.

## Layout of mining roadway of isolated island panel

### Layout form of mining roadway

**Figure 6:**
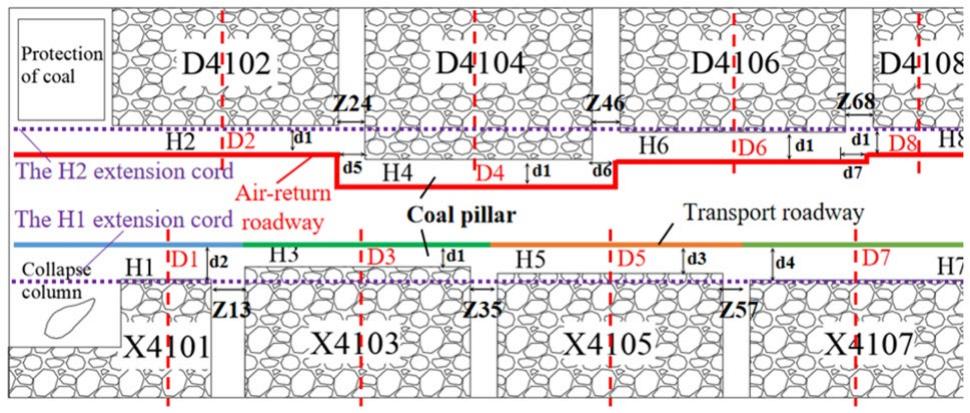
Layout of mining roadway in isolated island panel.

According to the distribution characteristics of boundary stress and plastic zone of goaf on both sides of isolated island panel, the layout of mining roadway in isolated island panel is characterized by seven typical characteristics. That is, d1–d4 is the width of the coal pillar between the mining roadway of the isolated island panel and the stopping line of each goaf, d5–d7 is the length of the mining roadway of the isolated island panel arranged in the width range of the coal pillar. The air-return roadway of the isolated island panel is arranged in a staggered way, and the transport roadway is arranged in a straight way, as shown in Fig. 6.

**Figure 7:**
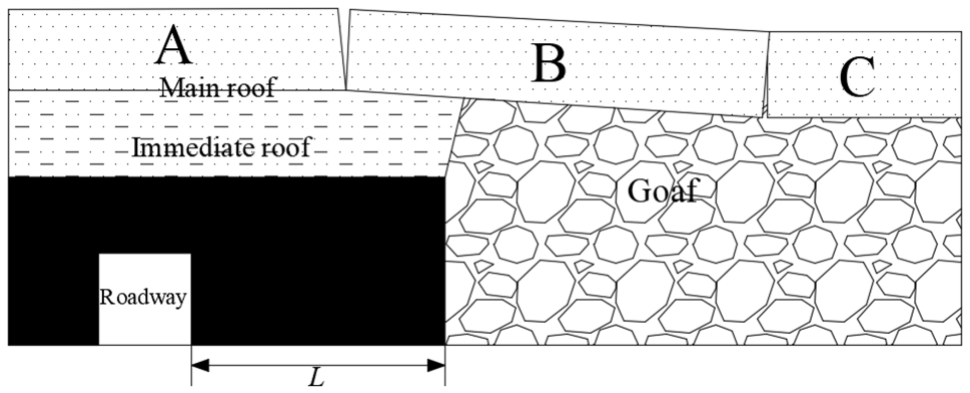
Coal pillar along goaf in isolated island panel.

After each goaf is stabilized, a stable “large structure” is formed in the top rock layer of the stopping line Hi_(i=1–8)_, so that the coal mass at the edge of the goaf has stress reduction zone, stress increase zone and original rock stress zone^11^. In order to reduce the stress of the surrounding rock of the mining roadway in the isolated island panel and ensure the stability of the mining roadway, the mining roadway should be arranged in the stress reduction zone at the edge of the goaf^12^. Therefore, the width of the coal pillar should be as small as possible to reduce the influence of the supporting stress on it. At the same time, it is necessary to ensure the stability of the bolt anchorage and comprehensively consider the deformation of the mining roadway. The details are shown in Fig. 7.

1 $$L = L_{1} + L_{2} + L_{3}$$

In the formula: *L* is the width of the coal pillar along the isolated island panel, m; *L*_*1*_ is the width of the stress reduction zone generated in the coal body at the edge of the goaf, 2.3–2.5 m; *L*_*3*_ is the effective length of the anchor bolt, taking 2.5 m ; *L*_*2*_ is the stability coefficient of coal pillar considering the thickness of coal seam, which is calculated by 0.2 × (*L*_*1*_ + *L*_*3*_). According to the above conditions, *L* = 5.76–6 m can be obtained.

Therefore, five schemes are designed for the width d1 of the coal pillar along the isolated island panel on the side of the air-return roadway: 6 m, 8 m, 10 m, 12 m and 14 m. The width d2–d4 of the coal pillar along the isolated island panel on the side of the transport roadway is determined by d1, and the length d5–d7 is the width of the coal pillar in each section. The mining roadway scheme of the isolated island panel is shown in Tables 2 and 3.

**Table 2:** Layout scheme of mining roadway in isolated island panel (Different widths of coal pillars).

The serial number	Different widths of coal pillars (m)				
d1	6	8	10	12	14
d2	18	20	22	24	26
d3	12	14	16	18	20
d4	18	20	22	24	26

**Table 3:** Layout scheme of mining roadway in isolated island panel (Length of mining roadway).

The serial number	Length of mining roadway (m)
d5	24
d6	24
d7	24

### Stress evolution of roadway surrounding rock during roadway excavation

#### Stress distribution law of surrounding rock of mining roadway within width range of parallel goaf

**Figure 8:**
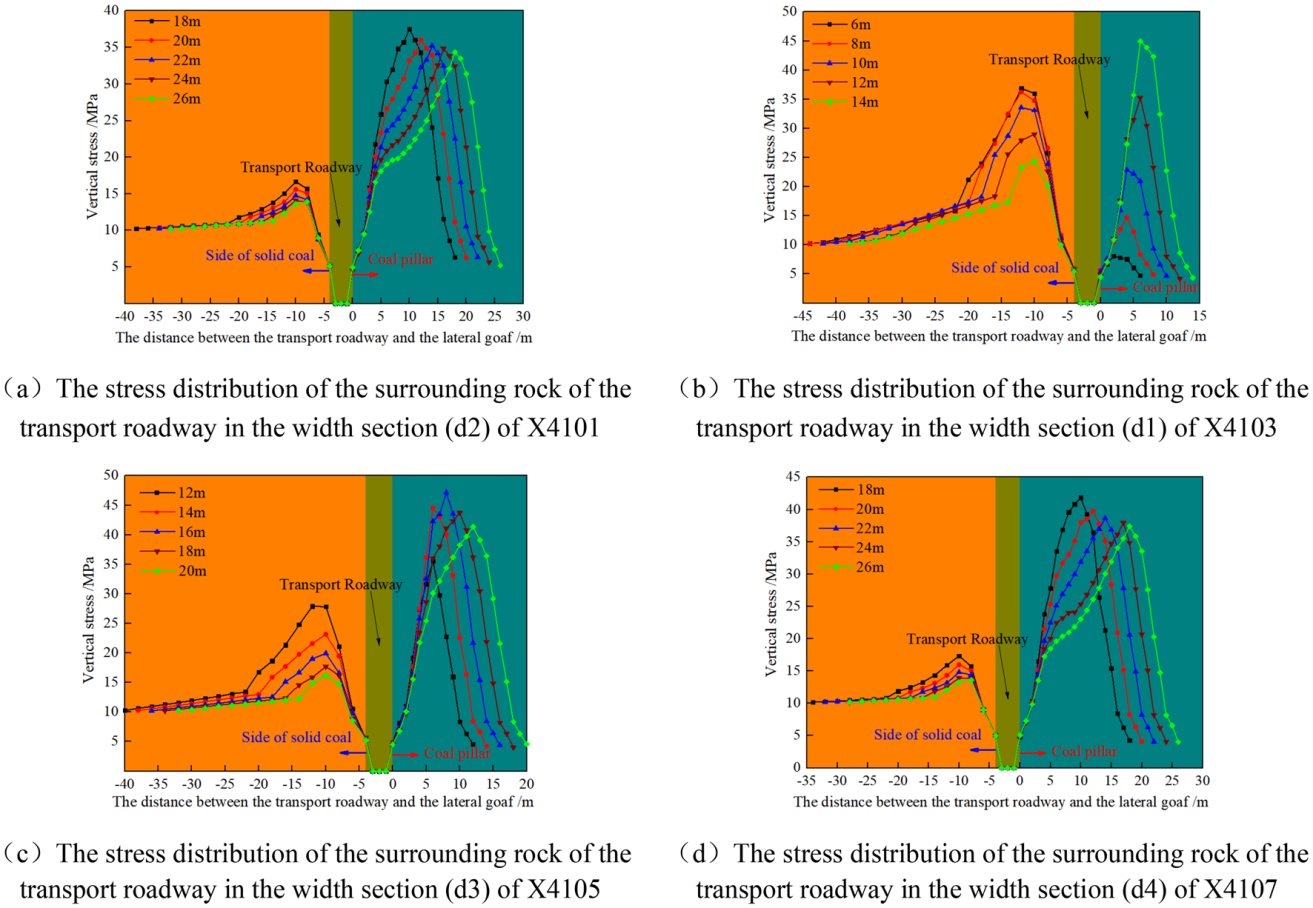
Stress evolution of surrounding rock of transport roadway in isolated island panel.

1. Within the width section parallel to the gob X410n_(n=1,3,5,7)_, when the transport roadway is arranged at different positions, the stress distribution of surrounding rock is shown in Fig. 8.

In Fig. 8a,d, as the width of the coal pillar d2 and d4 along the isolated island panel increases, the stress in the coal pillar along the isolated island panel decreases, and the high stress is distributed in the coal pillar along the air of the isolated island panel.

In Fig. 8b, with the increase of the width of the coal pillar d1 along the air of the isolated island panel, the stress in the coal pillar is rising. When the width of coal pillar d1 is 6 m, the peak stress is 8.07 MPa, which is lower than the original rock stress, and the coal pillar is in the state of failure. The peak stress is transferred from the side of the isolated island panel to the coal pillar in the goaf along the isolated island panel.

In Fig. 8c, with the increase of d3 width of the coal pillar in the island working face, the stress in the coal pillar in the island working face rises first and then decreases, and the high stress is distributed in the coal pillar in the isolated island panel.

**Figure 9:**
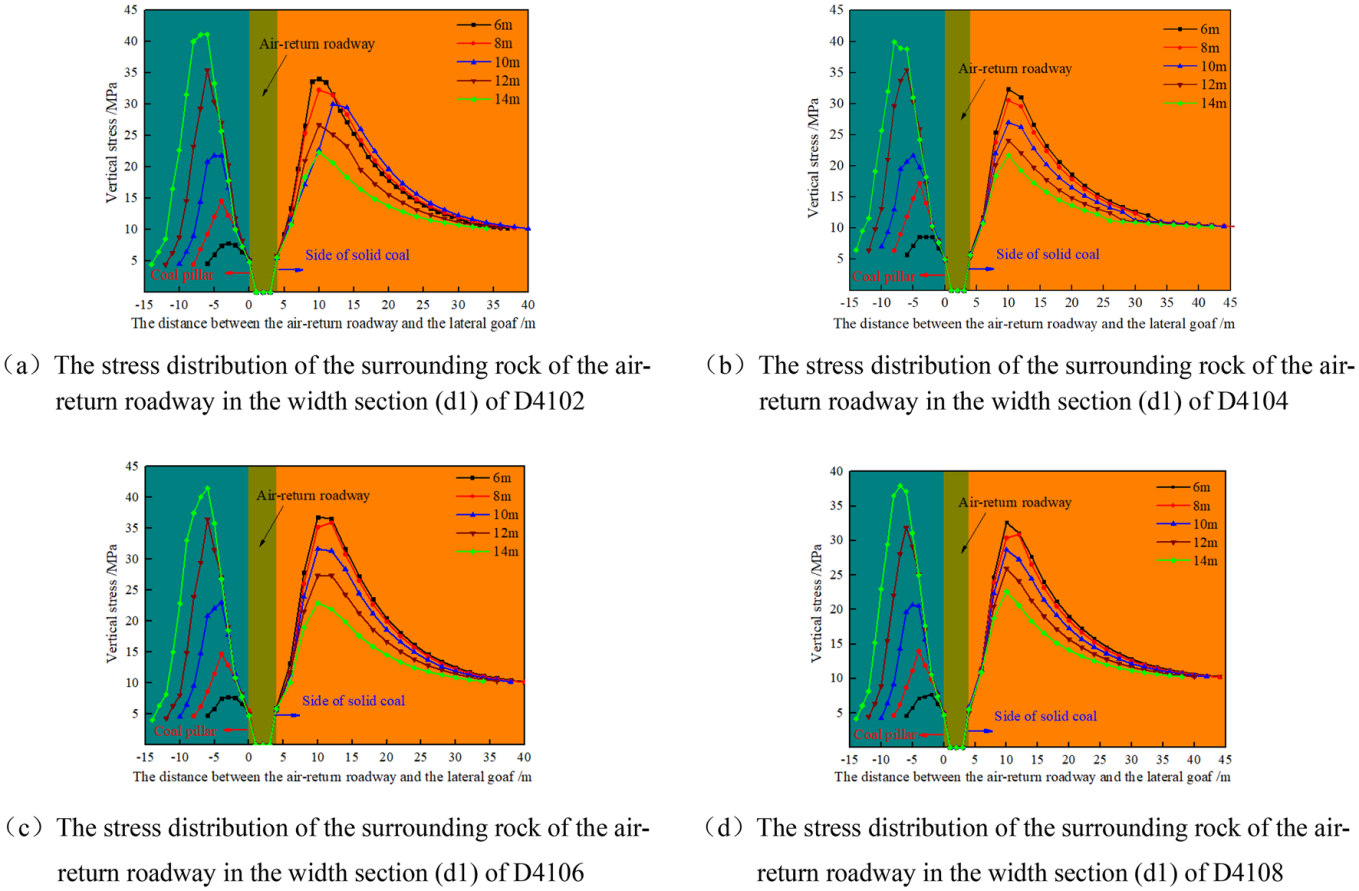
Stress evolution of surrounding rock of air-return roadway in isolated island panel.

(2) Within the width section parallel to the gob D410n_(n=2,4,6,8)_, when the air-return roadway is arranged at different positions, the stress distribution of surrounding rock is shown in Fig. 9.

According to Fig. 9a–d, with the increase of d1 width of the coal pillar of the isolated island panel on the side of the air-return roadway, the stress in the coal pillar of the isolated island panel is in an upward trend. When the width of coal pillar d1 is 6 m, its peak stress is 7.69–8.62 MPa, which is lower than the original rock stress, and the coal pillar is in the failure state. When the width of the coal pillar is 10–12 m, the peak stress is transferred from the side of the isolated island panel to the coal pillar.

The peak stress inside the coal pillar along the isolated island panel with different widths is shown in Table 4.

**Table 4:** Peak stress in the coal pillar of the isolated island panel.

Range of mining roadway	Relative spacing of the stopping line (m)	Peak stress at different coal pillar widths (MPa)					
X4101	0	d2	18 m	20 m	22 m	24 m	26 m
			37.5	36.01	35.23	34.84	34.33
X4103	12	d1	6 m	8 m	10 m	12 m	14 m
			8.07	14.71	22.87	35.26	44.97
X4105	6	d3	12 m	14 m	16 m	18 m	20 m
			35.51	44.53	47.12	43.74	41.36
X4107	0	d4	18 m	20 m	22 m	24 m	26 m
			41.81	39.77	38.63	37.94	37.41
D4102	0	d1	6 m	8 m	10 m	12 m	14 m
			7.79	14.57	21.76	35.42	41.14
D4104	30	d1	6 m	8 m	10 m	12 m	14 m
			8.62	14.76	21.66	35.4	41.06
D4106	6	d1	6 m	8 m	10 m	12 m	14 m
			7.77	14.73	22.99	36.44	44.46
D4108	2	d1	6 m	8 m	10 m	12 m	14 m
			7.69	13.99	20.63	31.84	37.92

The relative stopping line spacing is based on the stopping line H1/H2 extension line.

It can be seen from Table 3 that the peak stress in the coal pillar of the isolated island panel increases with the increase of the coal pillar width d1 in the width range of D4102, D4104, D4106, D4108 and X4103 in the goaf. The width of coal pillar d2, d3 and d4 in the isolated island panel belongs to the category of wide coal pillar lane protection. When the width of coal pillar is 16 m, it is the maximum width of vertical stress. When the width is less than or greater than 16 m, the vertical stress in coal pillar di_(2–4)_ shows a decreasing trend.

(3) Stress distribution law of surrounding rock of mining roadway in width section of coal pillar in parallel section.

**Figure 10:**
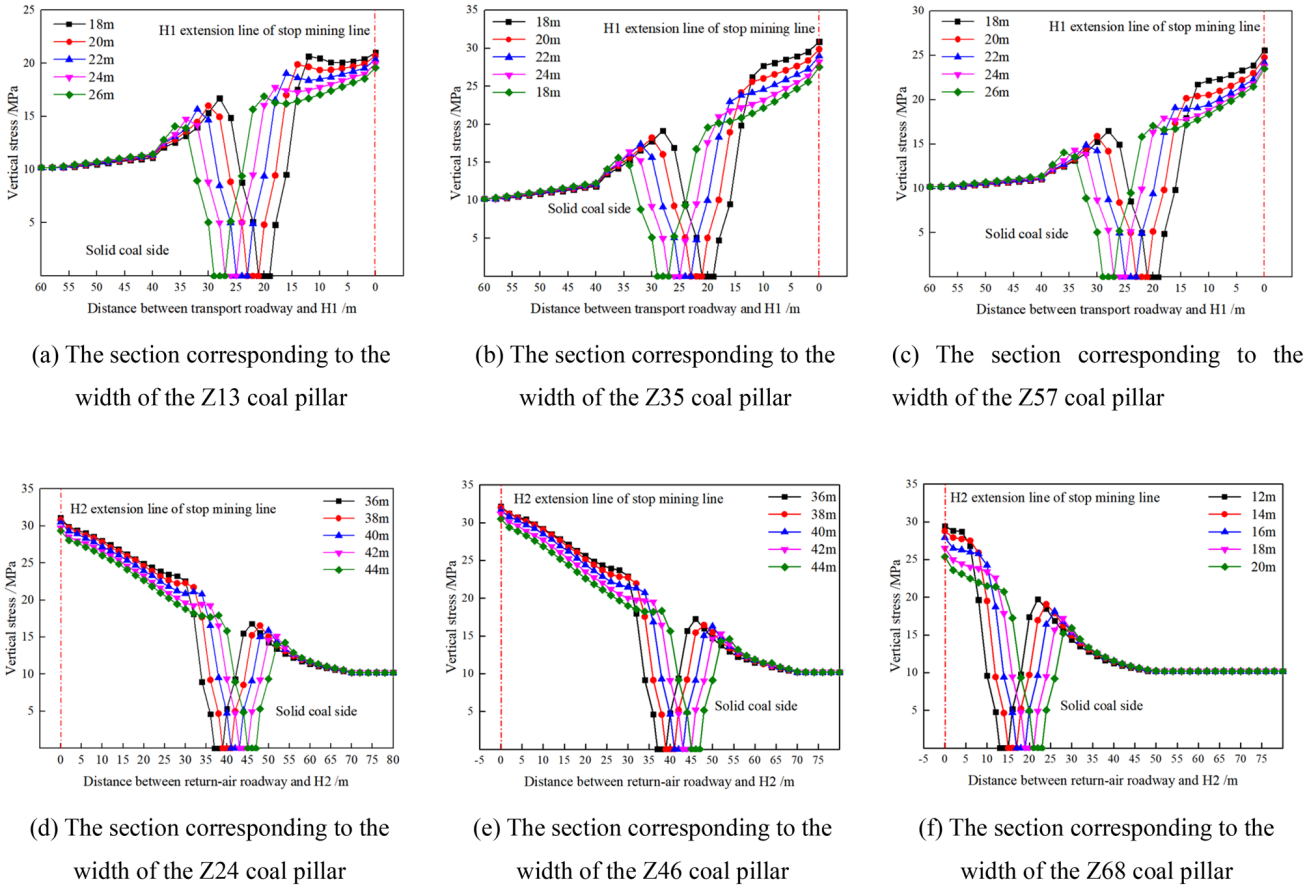
Stress distribution of surrounding rock of mining roadway under the width range of section coal pillar.

According to the scheme in Table 2 and the layout Fig. 6 of mining roadway, Fig. 10 shows the stress distribution law of surrounding rock of mining roadway corresponding to the width section of coal pillar Z_mn(mn=13, 35, 57, 24, 46, 68)_.

As shown in Fig. 10a–f, as the transport roadway and the air-return roadway on the isolated island panel move away from the H1/H2 extension line of the stopping line, the stress on both sides of the roadway decreases, and the high stress is distributed on the side of the stopping line. In the direction close to the H1/H2 extension line of the stopping line, the surrounding rock of the roadway is affected by the superimposed stress of the adjacent goaf, and the stress is concentrated. As the roadway moves away from the H1/H2 extension line of the stopping line, the vertical stress near both sides of the roadway tends to be symmetrically distributed, and the influence of the advance support pressure of the gob gradually weakens. Therefore, the mining roadway of the island working face far away from the lateral goaf and deeper into the island coal body can better maintain the stability of the surrounding rock, but it will increase the waste of coal resources.

In summary, when the coal pillar width d1 of the mining roadway corresponding to the width section X4103 of the goaf on the side of the air-return roadway is 6 m, the stress in the coal pillar is lower than the original rock stress. When the width d1 of the coal pillar in the isolated island panel is 10–12 m, the high stress is transferred from the side of the solid coal to the side of the coal pillar in isolated island panel. The width d1 of the coal pillar is greater than 12 m, and the coal pillar in the isolated island panel bears high stress. After comprehensive consideration, the coal pillar width d1 of the mining roadway corresponding to the width section X4103 of the goaf on the side of the air-return roadway is 6 m and 10 m for the mining study of the isolated island panel.

### Stress distribution of roadway surrounding rock during mining

**Figure 11:**
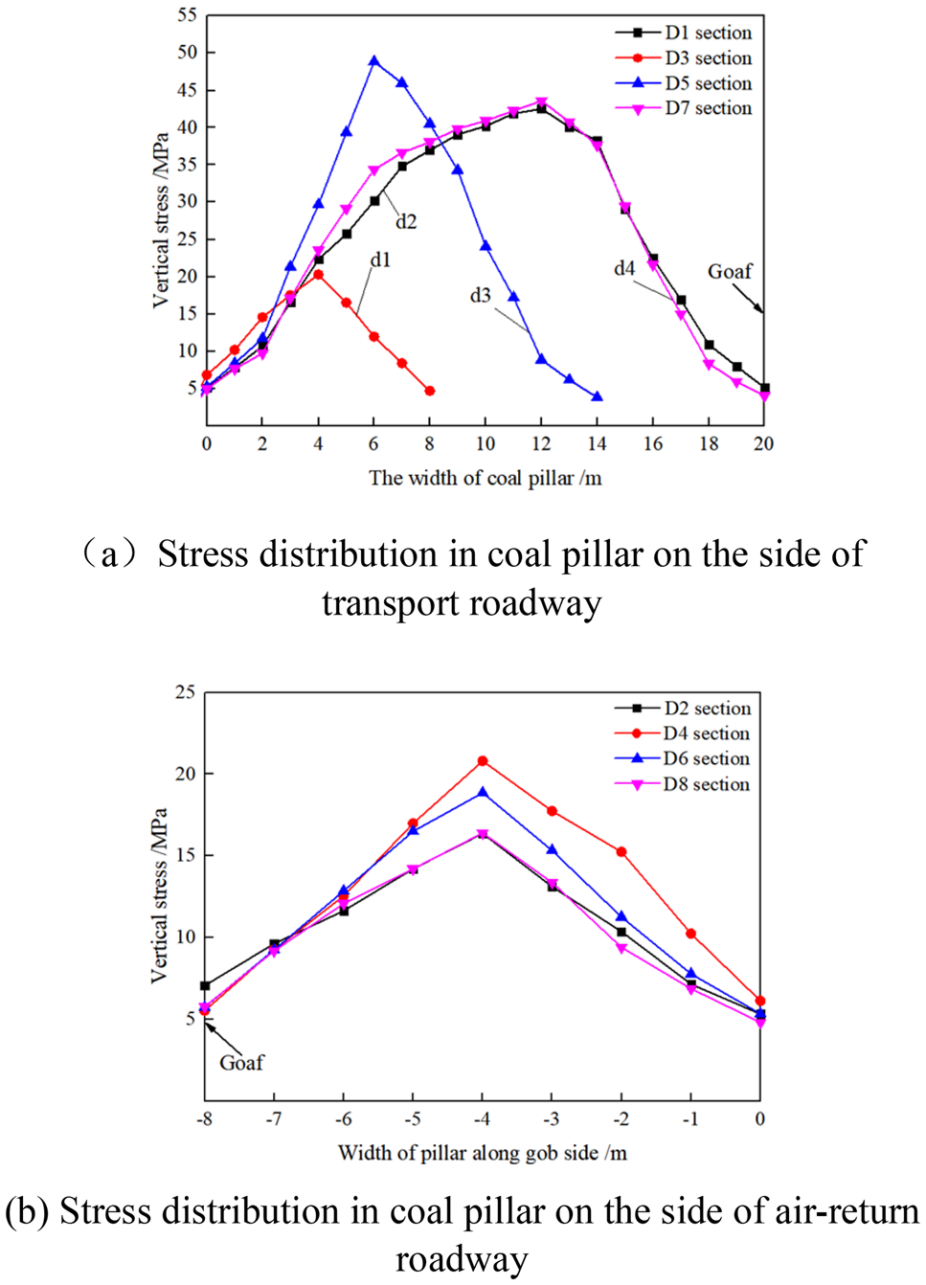
Stress in coal pillar when the isolated island pane is mined to different positions.

1. When the width of the coal pillar d1 of the island working face is 8 m, and the island working face is mined to the central position Di_(i=1–8)_ of each goaf, the stress distribution in the coal pillar of the isolated island panel is shown in Fig. 11.

As shown in Fig. 11a, when the isolated island panel advances to different positions, the stress in the coal pillar changes from unimodal distribution to semi-saddle distribution with the increase of the lateral coal pillar width of the transport roadway. With the progress of the isolated island panel, the peak stresses in the lateral coal pillar of the transport roadway during the process of section D1 → D3 → D5 → D7 are 42.53 MPa, 20.29 MPa, 48.85 MPa and 43.59 MPa, respectively.

As shown in Fig. 11b, the stress in the coal pillar side of air-return roadway is unimodal distribution. With the progress of the isolated island panel, the peak stresses in the lateral coal pillar of the air-return roadway during the process of section D2 → D4 → D6 → D8 are 16.36 MPa, 20.8 MPa, 18.85 MPa and 16.4 MPa, respectively.

**Figure 12:**
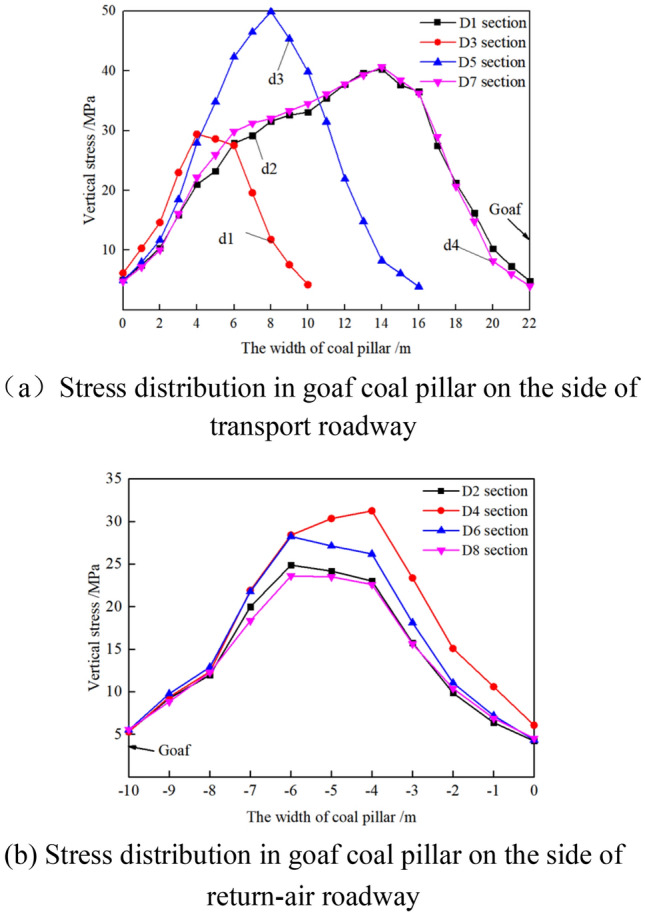
Stress distribution in the coal pillar when the isolated island panel advanced to different positions.

2. When the width of coal pillar d1 is 10 m and the isolated island panel is mined to Di_(1–8)_, the stress distribution in the coal pillar of isolated island panel is shown in Fig. 12.

As shown in Fig. 12a, when the isolated island panel advances to different positions, the stress in the coal pillar changes from unimodal distribution to semi-saddle distribution with the increase of the width of the lateral coal pillar in the transport roadway. In the process of the isolated island panel advancing from section D1 → D3 → D5 → D7, the peak stresses in the lateral coal pillar of the transport roadway are 40.26 MPa, 29.4 MPa, 49.87 MPa and 40.66 MPa, respectively.

As shown in Fig. 12b, the stress in the lateral coal pillar of the air-return roadway has a trapezoidal distribution. With the advance of the isolated island panel, the peak stresses in the lateral coal pillar of the air-return roadway are 24.88 MPa, 31.25 MPa, 28.23 MPa and 23.61 MPa, respectively, during the change of section D2 → D4 → D6 → D8.

In conclusion, when the goaf width d1 increases from 8 to 10 m, the stress in the coal pillar at the side of the air-return roadway increases, and the vertical stress distribution changes from acute triangular distribution to trapezoidal distribution. At the same time, the width of high stress bearing area increases, which increases the stability of coal pillar. However, the coal pillars d2, d3 and d4 on the side of transport roadway belong to the category of wide coal pillars, and their vertical stress distribution forms basically have no significant change.

### Displacement distribution of roadway surrounding rock during mining

**Figure 13:**
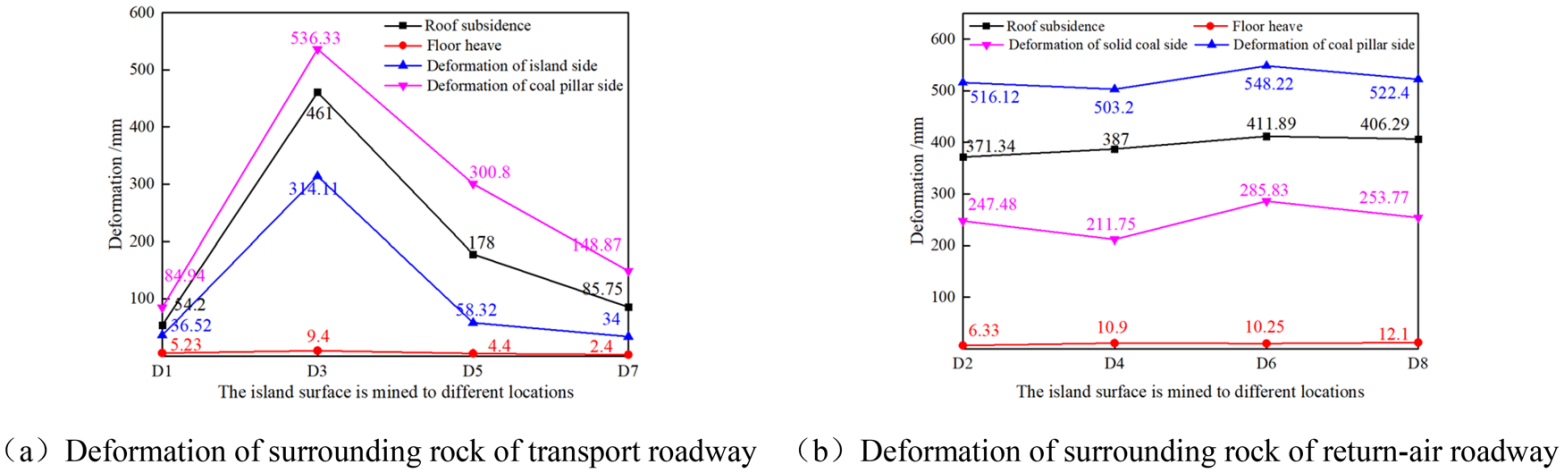
Deformation of surrounding rock of roadway during mining when the coal pillar (d1) is 8 m.

**Figure 14:**
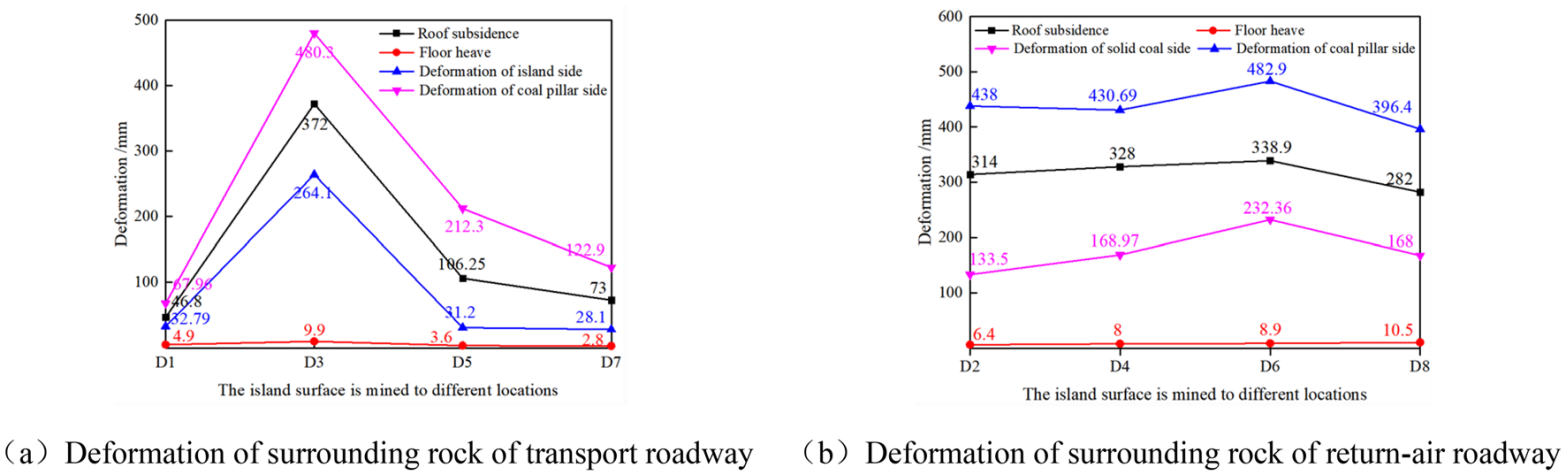
Deformation of surrounding rock of roadway during mining when the coal pillar (d1) is 10 m.

When the width of pillar d1 of the isolated island panel is 8 m and 10 m, it can be obtained that when the mining face is pushed to the roadway section Dn_(n=1–8)_, the deformation of surrounding rock of the roadway is shown in Figs. 13 and 14.

As shown in Fig. 13a, when the width of coal pillar d1 on the side of the mining roadway is 8 m and the mining face is advanced to D3 in the central position of goaf X4103, the deformation of surrounding rock of the roadway is the largest. Then it can be seen that the coal pillar wall convergence of the transport roadway is 536.33 mm, the solid coal wall convergence is 314.11 mm, the roof subsidence is 461 mm, and the floor heave is 9.4 mm. As shown in Fig. 13b, in the process of working face mining, the coal pillar wall bulge in the air-return roadway is generally more than 500 mm.

As shown in Fig. 14, when the width of coal pillar d1 on the side of the mining roadway is 10 m and the isolated island panel is advanced to D3 in the central position of goaf X4103, the deformation of surrounding rock of the roadway is the largest, the convergence of coal pillar wall of the transport roadway is 480.3 mm, the convergence of solid coal side is 264.1 mm, the roof subsidence is 372 mm, and the floor heave is 9.9 mm. As shown in Fig. 14b, during the mining process of the isolated island panel, the coal pillar side convergence of the air-return roadway is generally less than 480 mm..

In summary, on the basis of the stress, the deformation and recovery improvement of the surrounding rock of the mining roadway in the isolated island panel, on the air-return roadway side of the isolated island panel, the air-return roadway is arranged in a staggered way, and the coal pillar width d1 of the isolated island panel is selected as 10 m, and the length of d5–d7 is the width of the coal pillar in each section. On the side of the transport roadway on the isolated island panel, the transport roadway is arranged straight, and the distance between the transport roadway and the stopping line of goaf X4103 is 8 m.

## Safe mining and support technology in strong dynamic pressure area of the isolated island panel

### Strong dynamic pressure of the isolated island panel affects the process

**Figure 15:**
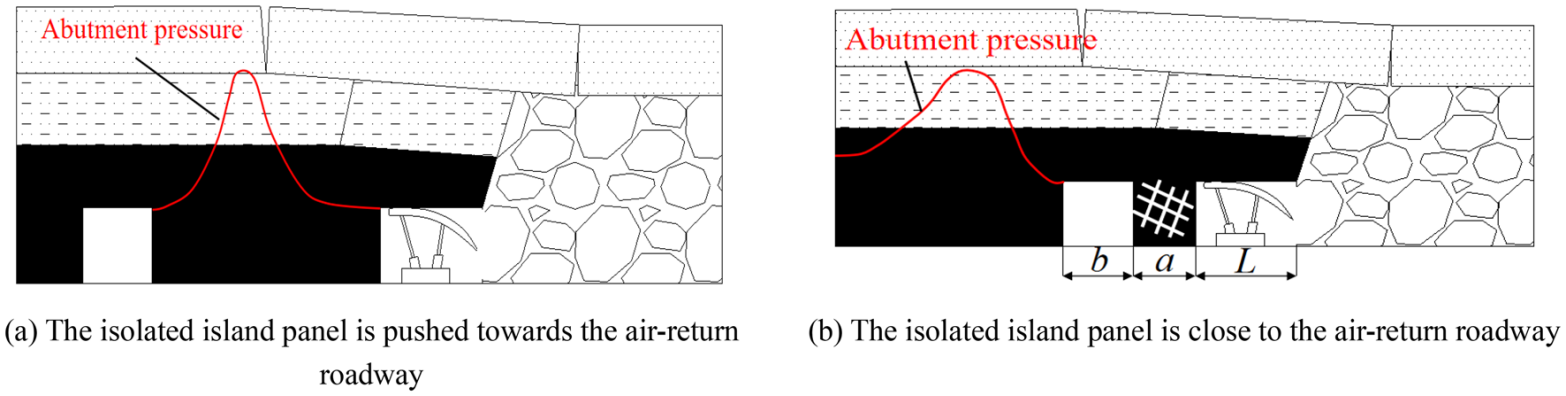
Advancing process of the isolated island panel in the direction of the air-return roadway.

In the mining process of isolated island panel, it is frequently affected by strong dynamic pressure, which brings great hidden dangers to the safety production of mine^13–15^. It is easy to induce strong dynamic pressure when the working face is close to the air-return roadway with staggered arrangement. Since the air-return roadway cuts the coal seam of the isolated island panel to form a coal pillar, when the mining face is close to the air-return roadway with staggered arrangement (Fig. 15a), the width of the coal pillar between them gradually decreases and the stress concentration on the coal pillar increases continuously, which is easy to induce rock burst, dynamic pressure of stope support and storm of the air-return roadway. However, when the working face is close to the air-return roadway with staggered arrangement, the coal pillar is very broken and almost has no bearing capacity. At this time, the roof control distance of the hydraulic support in the working face changes from *L* to (*L* + *a* + *b*) (Fig. 15b), which leads to a sudden increase in the support load and is easy to induce the stope frame collapse accident. In Fig. 15b, L is the top control distance of the support; *a* is the width of broken coal pillar; *b* is the width of staggered air-return roadway Therefore, this paper simulates the stress evolution near the working face and the influence range of abutment pressure in the advancing process of section pillar Z24 and Z46.

**Figure 16:**
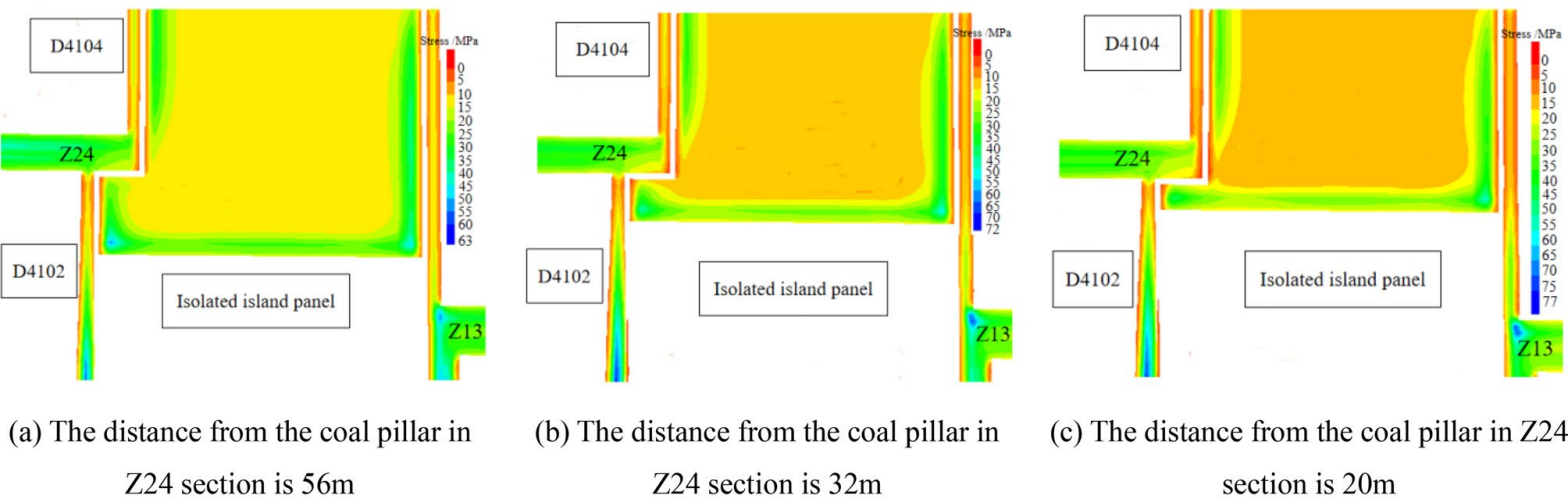
Abutment pressure distribution of coal body (Z24).

1. As shown in Fig. 16, the vertical stress distribution when the isolated island panel gradually approaches coal pillar Z24.

As can be seen from Fig. 16, with the advance of the working face, the working face is closer to the coal pillar Z24, and then the stress at the corner of solid coal changes. When the distance between the working face and coal pillar Z24 is 56 m, the leading abutment pressure begins to spread to the coal pillar Z24, and then the working face begins to enter the impact danger zone. The peak stress of the working face at the air-return roadway side is distributed at the solid coal corner of the working face, and its peak stress is 52.26 MPa. As the working face continues to advance, when the distance between the working face and coal pillar Z24 is 32 m, the peak stress at the solid coal corner on the air-return roadway side of the working face is 48.1 MPa. When the distance between the working face and coal pillar Z24 is 20 m, the peak stress at the solid coal corner on the air-return roadway side of the working face is 45.5 MPa. When the stress in the coal body between the working face and coal pillar Z24 begins to decline, it indicates that the coal body has begun to yield and the bearing capacity of the coal body gradually decreases. At this time, the isolated island panel begins to enter the danger zone of frame compression. Therefore, when mining in the working face and coal pillar, pressure relief should be carried out on the solid coal side of the air-return roadway of the isolated island panel.

**Figure 17:**
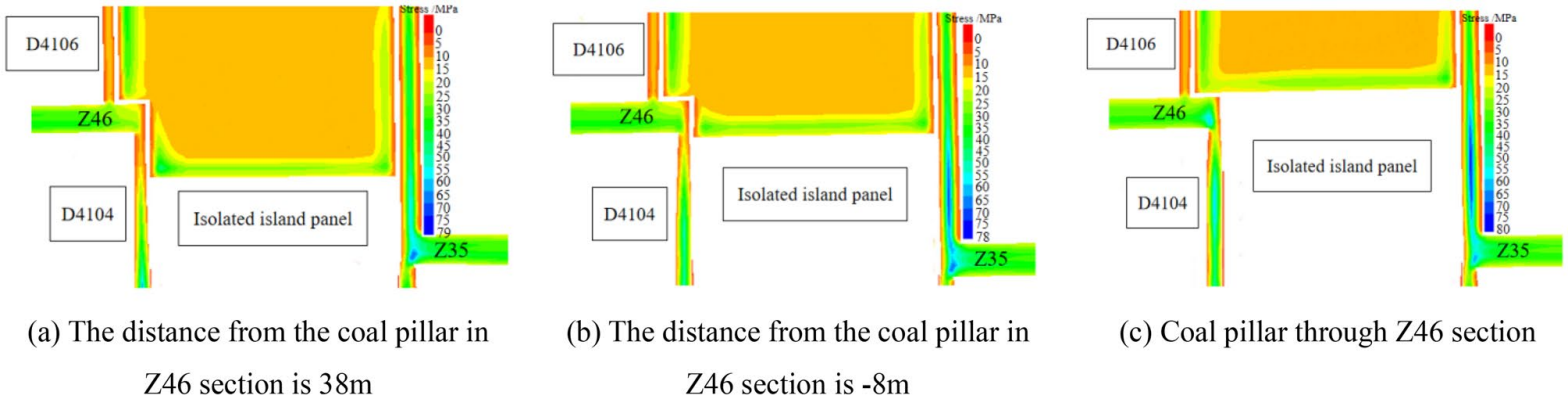
Abutment pressure distribution of coal body (Z46).

2. As shown in Fig. 17, the vertical stress distribution when the isolated island panel gradually approaches coal pillar Z46.

According to Fig. 17, when the distance between the isolated island panel and coal pillar Z46 is 38 m, the leading abutment pressure of the isolated island panel runs through the area of coal pillar Z46, and the peak stress of the working face is distributed at the solid coal corner on the side of the air-return roadway of the working face, and its peak stress is 48.3 MPa. When the working face advances to the Z46 section of the coal pillar and reaches -8 m, the peak stress of the air-return roadway side of the working face near the solid coal is 27.8 MPa, and the peak stress of the side near the coal pillar Z46 is 46.6 MPa. When the working face passes through the Z46 section of the coal pillar completely, the peak stress is distributed at the solid coal corner of the air-return roadway of the working face, and the peak stress is 30.1 MPa. When the working face passes through the Z46 section of coal pillar, the peak stress distribution near the air-return roadway side of the working face is a jump type: solid coal side of the working face → Z46 side of the section coal pillar → solid coal side of the working face. Therefore, the air-return roadway parallel to the width of pillar Z46 has no impact hazard.

### Rock burst prevention measures

**Figure 18:**
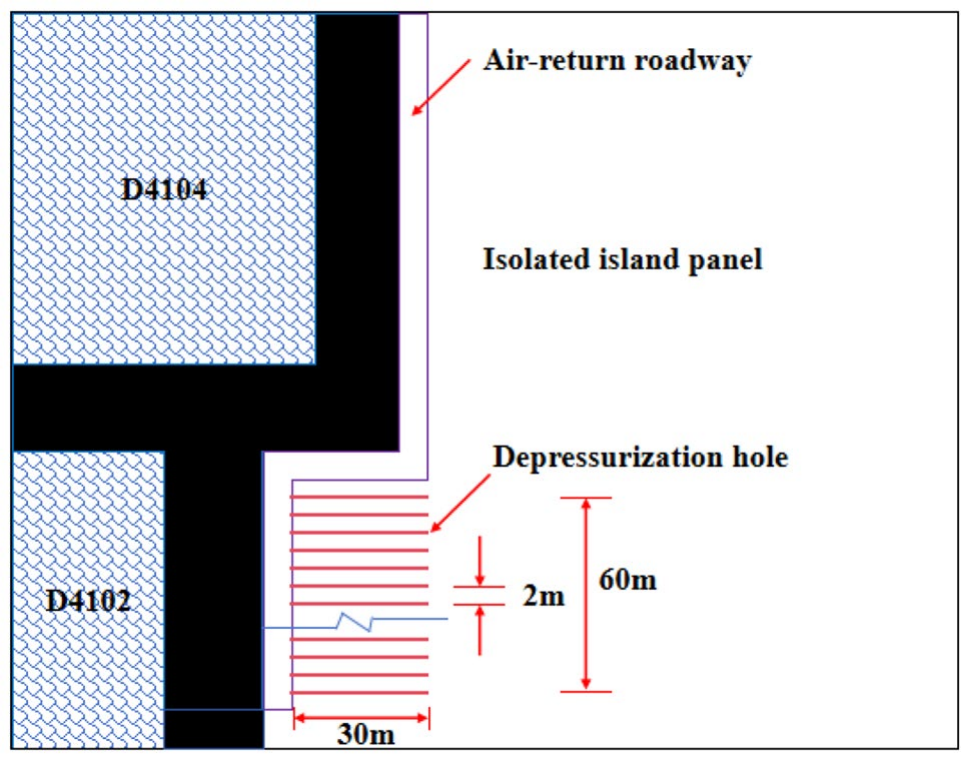
Layout of pressure relief drill hole.

In order to ensure the safe passage of the isolated island panel through the strong dynamic pressure zone, pressure relief treatment technology is adopted^16–22^. According to the engineering analogy method, in the advancing direction of the isolated island panel, the pressure relief treatment is carried out in the area where the advance working face is 200 m and the distance from the coal pillar Z24 is 60 m. The diameter of the pre-relief drilling hole is 150 mm, the hole distance is 2 m, and the depth is 30 m. The pressure relief drilling arrangement is shown in Fig. 18.

### Determination of surrounding rock control parameters

**Figure 19:**
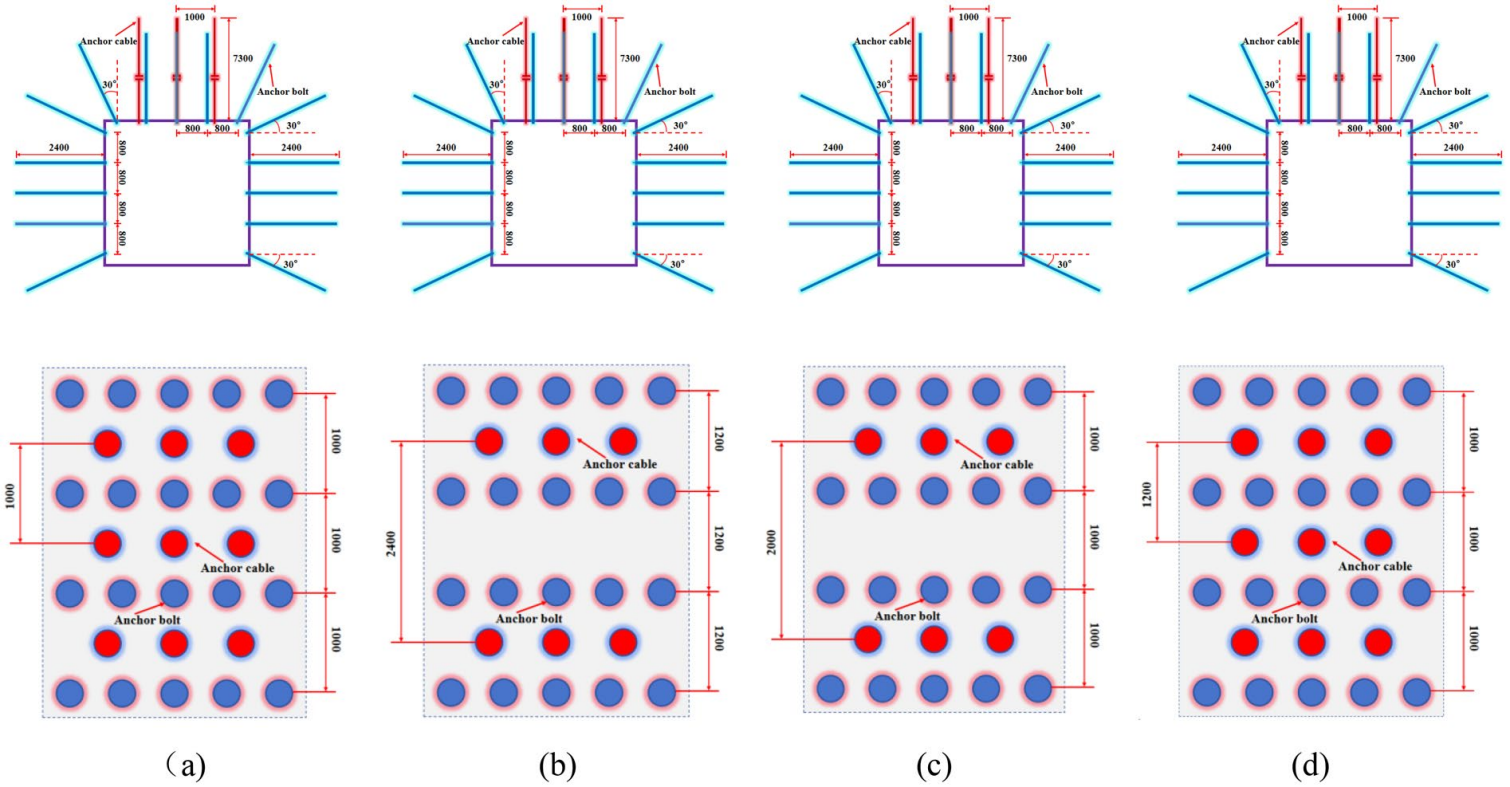
Analysis of mining roadway support system.

Based on the dynamic design method and engineering analogy method, combined with the technical conditions of the mine, considering the feasibility and convenience of construction, the mining roadway of the isolated island panel is divided into supporting scheme. The main supporting parameters of the roadway cross-section of the isolated island panel are as follows: Five rebar bolts of up to 22 mm × 3500 mm are arranged in the roof; Five glass reinforced plastic bolts of *φ*22mm × 2400 mm were arranged on each side of the roadway. The roof is arranged with three 19-strand steel anchor cables (*φ*17.8 mm × 7300 mm), and 10# diamond metal mesh and 3.5 mm T-shaped steel belt are laid. The mining roadway is controlled in four areas: (a) Support structure of transport roadway parallel to the width section of goaf X4103; (b) Support structure of transport roadway arranged in width section of goaf X4103; (c) The support structure of the air-return roadway parallel to the width section of the goaf; (d) The air-return roadway is parallel to the support area of the coal pillar section, and the specific supporting structure is shown in Fig. 19.

## Engineering practice of isolated island panel

In order to study the rationality of the control parameters of the surrounding rock of the isolated island panel, the surface displacement(roof, floor, coal pillar side, solid coal side) of surrounding rock of the roadway was continuously monitored (two measuring points).

**Figure 20:**
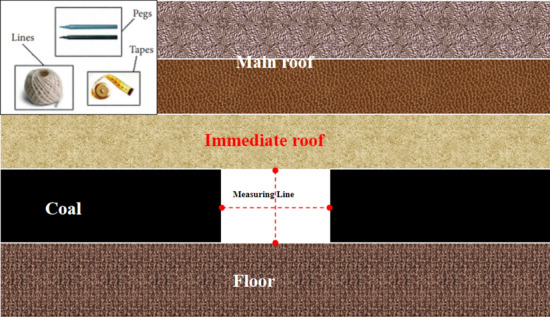
Layout of the measurement station.

**Figure 21:**
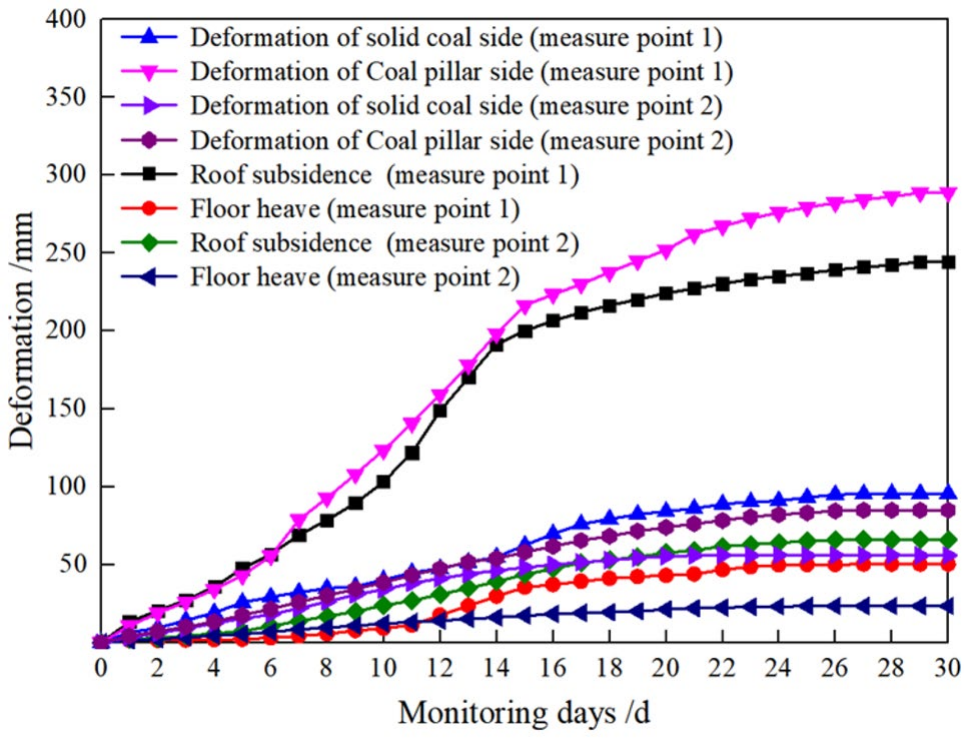
Monitoring chart of mining roadway during roadway excavation.

The monitoring point 1 was the regional roadway of the width section D4102 of goaf, and the monitoring point 2 was the roadway of the width area Z24 of coal pillar, as shown in Figs. 20 and 21. The surrounding rock deformation of the roadway remains stable after 23–25 days. The roadway deformation at measuring point 1 is as follows: the roof subsidence is 244.6 mm, the floor heave is 50.3 mm, the convergence of solid coal side is 95.7 mm, and the convergence of coal pillar side is 288.7 mm. The roadway deformation at the 2 measurement points is as follows: the roof subsidence is 66 mm, the floor heave is 23.5 mm, the convergence of solid coal side is 56 mm, and the convergence of coal pillar side is 85 mm.

## The conclusion

1. According to the differences in the distribution morphology of the goaf on both sides of the isolated island coal pillar, the mining characteristics of the goaf boundaries on both sides of the isolated island coal pillar are obtained. The peak stress at the end of each coal pillar is 35–40 MPa. The coal pillar bears high stress, and the stress zone of the original rock moves to the end of the coal pillar. There is a plastic zone of 8–20 m at the corner of the end of each coal pillar.
2. During roadway excavation in the isolated island panel, the peak stress in the coal pillar of the isolated island panel increases with the increase of the coal pillar width d1. The width of coal pillar d2, d3 and d4 in the isolated island panel belongs to the category of wide coal pillar roadway. When the width of coal pillar is 16 m, it is the maximum width of vertical stress. When the width is less than or greater than 16 m, the vertical stress in coal pillar di(2, 3, 4) shows a decreasing trend. As the mining roadway parallel to the width section of the coal pillar is far away from the H1/H2 extension line of the stopping line, the vertical stress near both sides of the roadway tends to be symmetrically distributed, and the influence of the advance support pressure of the goaf is gradually weakened.
3. According to the distribution characteristics of stress and plastic zone of the isolated island panel at the boundaries of multiple goaf, the layout mode and principle of roadway are proposed. The roadway must avoid the high stress area of the goaf boundary and ensure the deformation of the mining roadway. Four typical coal pillar widths and three mining roadway layout lengths are determined.
4. According to the disaster mechanism of strong dynamic pressure zone, the corresponding safe mining technology is put forward. In order to ensure the safe mining of the working face, it is proposed that the roadway of the isolated island panel is divided into four areas of surrounding rock control.

## Data Availability

All data used to support the findings of this study are included within the article.
